# Action Fluency in Parkinson’s Disease: A Mini-Review and Viewpoint

**DOI:** 10.3389/fnagi.2021.778429

**Published:** 2021-11-25

**Authors:** Claudia Gianelli, Carlotta Maiocchi, Nicola Canessa

**Affiliations:** ^1^Cognitive Neuroscience Laboratory of Pavia Institute, Istituti Clinici Scientifici Maugeri IRCCS, Pavia, Italy; ^2^IUSS Cognitive Neuroscience (ICON) Center, Scuola Universitaria Superiore IUSS, Pavia, Italy

**Keywords:** Parkinson’s disease, action fluency, action processing, embodied cognition, movement disorders

## Abstract

Increasing evidence shows that the typical motor symptoms of Parkinson’s disease (PD) are often accompanied, if not preceded, by cognitive dysfunctions that are potentially linked to further complications of the disease. Notably, these cognitive dysfunctions appear to have a significant impact in the domain of action processing, as indicated by specific impairments for action-related stimuli in general, and verbs in particular. In this mini-review, we focus on the use of the action fluency test as a tool to investigate action processing, in PD patients. We discuss the current results within the embodied cognition framework and in relation to general action-related impairments in PD, while also providing an outlook on open issues and possible avenues for future research. We argue that jointly addressing action semantic processing and motor dysfunctions in PD patients could pave the way to interventions where the motor deficits are addressed to improve both motor and communicative skills since the early disease stages, with a likely significant impact on quality of life.

## Introduction

Increasing evidence shows that the typical motor symptoms of Parkinson’s disease (PD) are often accompanied, if not preceded, by cognitive dysfunctions that are potentially linked to further complications of the disease, including developing dementia (Auclair-Ouellet et al., 2017). Notably, these cognitive dysfunctions appear to have a significant impact in the domain of action processing, as indicated by specific impairments for action-related stimuli in general, and verbs in particular (Cardona et al., 2013). While it is widely acknowledged that processing verbs is generally more difficult compared to nouns, regardless of their semantic content, evidence in the domain of embodied cognition suggests that PD patients might show a more specific impairment in this sense (Boulenger et al., 2008). In this framework, linguistic processing is thought to be grounded in sensorimotor processes in such a way that processing bodily action-related verbs or sentences (e.g., “to kick” or “I kick”), as well as concrete nouns (e.g., “ball”), is accompanied by sensory and motor activations congruent with their semantic content. Increasing evidence in healthy participants supports this view, both at the neural and the behavioral level (Pulvermüller, 2018), and more recently it has been shown that action-related verb processing, and to a lesser extent the comprehension of concrete nouns might be specifically impaired in PD patients, with progressive decay of motor skills accompanied by category-specific linguistic deficits (Bocanegra et al., 2015). Notably, it has been shown that these category-specific deficits emerge in a wide range of tasks (Cardona et al., 2013; Gallese and Cuccio, 2018). When action naming tasks are considered, existing studies generally report worse performance for PD compared to healthy controls (Péran et al., 2009) and other patient groups (e.g., Alzheimer’s disease; Rodríguez-Ferreiro et al., 2009). As to verb generation, PD patients typically show specific deficits for this category (Crescentini et al., 2008) while performing similarly to controls when nouns are considered (Péran et al., 2003). Interestingly, similar impairments have been shown not only when action-related words, and verbs in particular, are tested in isolation, but also when embedded in sentences (Cardona et al., 2014) and naturalistic texts (García et al., 2018).

It has thus been suggested that performance in tasks involving both comprehension and production might detect early impairments of the fronto-striatal loop, thus allowing to investigate how dopaminergic medication and other mechanisms might come into play to compensate for the progressive decay of action processing skills (Auclair-Ouellet et al., 2017). Furthermore, as these symptoms might severely impact communication abilities even in the first stages of the disease (Dadgar et al., 2013), early detection and full characterization of these deficits are crucial to improve the quality of life of these patients and design effective interventions. It is thus crucial to identify those tests that might be routinely used as screening tools allowing an early identification of specific action-related deficits and implementation of targeted interventions.

This review focuses on one example in this sense, i.e., the well-known action fluency task (Piatt et al., 1999a,b). By instructing participants to verbally report as many different actions they think people can do (e.g., “eat,” “smell”) in 1 min (Auclair-Ouellet et al., 2020), this test gives a fast and easy access to action word production and global action processing abilities. In this article, we will first review evidence from action fluency tests in general, then specifically address the use of this tool in PD patients within the embodied cognition framework and discuss the existing evidence providing a possible outlook for future research.

## The Action Fluency Test

Action fluency tests were originally implemented (Piatt et al., 1999a,b) to assess whether the ability to generate verbs in the absence of prompting stimuli reflects a unique aspect of executive functioning typically neglected both by action naming tasks (i.e., verb retrieval, as opposed to object naming/noun retrieval), and by well-established fluency tasks based on prompting stimuli such as first letter or semantic domain (i.e., lexical and categorical fluency, respectively). Action fluency was indeed expected to tap both the generative-executive demands inherent in fluency compared with naming tasks, and the specific integrative-executive demands intrinsic to retrieving verbs (Grossman, 1998) compared to nouns or categories (Piatt et al., 1999a,b).

Several studies have provided multifaceted support to the possible uniqueness of verb generation, in terms of neuro-cognitive processing and sensitivity to pathology. First, data from healthy controls showed that its performance is significantly, but moderately, related to other executive metrics, and unrelated to measures of semantic or episodic memory (Piatt et al., 1999b) which suggests that action fluency taps unique abilities within the realm of executive functioning. Supporting this notion, a disproportionate impairment of action, compared with lexical and semantic, fluency was first observed in demented Parkinson’s patients, compared both with non-demented patients and with elderly controls (Piatt et al., 1999a). Moreover, the lack of association between action fluency and dementia severity was suggestive of its specific sensitivity to the progressive fronto-striatal dysfunction of Parkinson’s disease. While the authors of these studies generically suggested that action fluency is a valid measure of executive and language functions (e.g., Piatt et al., 2004), more recently its semantic-conceptual requirements have been interpreted within the embodied cognition framework, i.e., in terms of neural action representations generated from, and tightly connected with, sensorimotor experiences (Salmazo-Silva et al., 2017). A prominent impairment of action fluency in Parkinson’s disease might thus reflect the adverse effect of basal ganglia damage on motor-language coupling (Melloni et al., 2015).

## Action Fluency in Parkinson’s Disease

Differently from action naming, action fluency tasks require participants to produce verbs in absence of any visual aid. Piatt et al. (1999a) first tested this ability in absence of prompting stimuli (i.e., action fluency) in PD patients with and without dementia and in healthy controls, showing that this task is instrumental in differentiating between the two patient populations. Notably, other fluency tasks (e.g., lexical and categorical) often failed to account for such a difference, supporting the notion that appropriate action fluency tasks specifically tap into the disease’s peculiar pathophysiology and deficits in executive functioning. These first results were supported by further data from the same group (Piatt et al., 2004) on healthy elderly controls. In this case, not only did the action fluency task prove its validity as a measure of executive functions, but the two measurements were significantly correlated, while appearing unrelated to semantic and episodic memory.

In this sense, an appropriate assessment of action fluency in PD patients necessarily requires disentangling these effects from those derived from other linguistic tasks (e.g., naming). Notably, direct comparisons between performance in fluency and naming tasks provided conflicting results. On the one hand, a few studies found no significant evidence of a clear distinction between action fluency and action naming (Rodríguez-Ferreiro et al., 2009). Bocanegra et al. (2017) compared PD participants with and without mild cognitive impairment, as well as healthy volunteers, in a picture-naming task revealing that PD-MCI patients were selectively impaired in processing action verbs semantically implying a high level of motion. In line with these results, Salmazo-Silva et al. (2017) tested the variability of PD patients’ impairments across a range of tasks focusing on action and object lexical and semantic processing with varying cognitive demands (verbal fluency, naming, and semantic association). Notably, while PD patients performed worse than controls in naming and association tasks, this did not hold for action fluency. The authors addressed these potentially conflicting results by highlighting the inherent confound posed by individual differences in the educational level. This factor might not only play a direct role in affecting performance in this specific task, but also in the preservation of executive functioning.

On the other hand, impairments in action fluency appeared to be more evident in comparison with lexical and semantic fluency. Rodrigues et al. (2015) directly compared action fluency scores with those derived from traditional fluency tests in PD in non-demented PD patients and healthy age- and education-matched controls. All participants were administered tests of letter, semantic, and action verbal fluency with significant differences between the two groups emerging only in the latter. Crucially, the fact that this specific deficit of verb vs. noun production appeared in PD patients without dementia – thus before any clear sign of cognitive impairment – supports the idea that a selective disruption in this domain is linked to fronto-striatal circuitry dysfunction (Fine et al., 2011). Notably, it has been proposed (Bocanegra et al., 2015) that while linguistic and semantic deficits in PD, specifically those related to action-verb production and action semantics, emerge very early during the first stages of the disease in absence of cognitive or executive dysfunctions, this is not the case for object semantics. Indeed, measures of executive functioning significantly predict impairments in this domain (Bocanegra et al., 2015). Similar results were obtained when testing PD patients without dementia and healthy controls longitudinally across several cognitive scales, as well as semantic, letter, and action fluency tasks (Signorini and Volpato, 2006). In this study, PD patients showed a consistent action fluency deficit in absence of other relevant cognitive disorders, thus supporting the notion that poor performance in this task can be interpreted as a sign of fronto-striatal damage.

Further evidence of a strong relationship between verb processing and motor functions was increasingly provided also by neuroimaging studies (York et al., 2014). By exploring the neural correlates of action-verb representations in PD with functional magnetic resonance imaging (*f*MRI), one of the early studies in this field (Péran et al., 2009) found a significant correlation between severity of the motor deficit (Unified Parkinson’s Disease Rating Scale – UPDRS score) and brain activity during action verb generation in the pre- and post-central gyri bilaterally, left frontal operculum, left supplementary motor area, and right superior temporal cortex. More recently, Auclair-Ouellet et al. (2020) coupled the action fluency test with *f*MRI in PD patients to shed light on the relationship among these measures and personal characteristics, disease factors, cognition, and neural activity. While the action fluency scores remained independent of linguistic measures, this test allowed o identify a subset of patients with peculiar sex, age, global cognitive profile, executive function scores, and brain activity. Notably, when dividing patients into two subgroups characterized by normal and poor action fluency performance, the latter group had worse scores in both the cognitive and executive function domains, as well as decreased activity in fronto-temporal regions. In line with other studies (Abrevaya et al., 2016), this finding suggests that, to compensate for movement disorders, action-verb processing, and possibly action processing in general, may rely on less efficient non-motor semantic circuits. This proposal would thus imply the co-occurrence of preserved semantic knowledge and alteration in the neuro-cognitive mechanisms mediating the use of linguistic-induced motor brain activity to gain more efficient access to action semantics. Crucially, data from brain connectivity analyses showed that patients and controls shared similar patterns when processing nouns. However, this was not the case for action-related words, with patients appearing to rely more strongly than controls on temporal areas involved in amodal semantics, likely representing a compensatory mechanism for the reduced availability of more direct and efficient motor routes. Supporting evidence in this sense came also from studies investigating the effect of medication. It has indeed been shown (Herrera et al., 2012) that, when testing PD patients on- and off-dopamine treatment, patients in the off-state produce significantly fewer verbs in the action fluency task compared to controls, with significant differences also in the frequency of the produced words.

Finally, additional converging evidence was recently provided by a direct comparison of action verb knowledge in patients with PD and amyotrophic lateral sclerosis (ALS), two pathologies characterized by different anatomical substrates and clinical manifestations (Cousins et al., 2018). Patients were classified as having high or low motor impairments based on disease-specific functional scales and were tested on a verb production task focusing on body-related verbs (i.e., where the body is either as the agent of the action or as the theme). Interestingly, PD patients showed a similar impairment in production for all verb types, regardless of the role of the body, whereas ALS participants showed a specific dissociation between agent- and theme-related body verbs.

## Outlook and Future Developments

We reviewed the use of action verbal fluency tests in investigating action processing in PD patients. While the existing literature on PD is still relatively limited, this test already showed its potential to highlight different facets of action processing impairments in this population, even compared to other fluency or naming tasks. Despite promising results, however, several open issues remain.

First, there is conflicting evidence concerning how performance in this test relates to naming tasks and other fluency assessments, as well as action processing in general. While this seems partially explained by the nature of the test, requiring participants to produce as many verbs referring to actions as possible, further research should aim to place action verbal fluency tests within a broader assessment of action semantic processing. In this sense, performance in this specific task should be evaluated not only with respect to scores in similar tests, but also against patients’ behavioral performance in tasks specifically designed to address motor-language coupling (Melloni et al., 2015). Preliminary evidence using an adapted version of the action-sentence compatibility effect (Cardona et al., 2014) suggests that this might indeed be a promising avenue (but see Morey et al., in press regarding limitations of this specific task).

With the aim of a more comprehensive characterization of the global profile of action-related impairments, research has so far mainly focused on the relationship between action fluency measures and other linguistic and executive function metrics pointing to a marked independence of this measure from scores in other tasks. However, future investigations will also have to consider a possible role of individual differences in driving action fluency results. As already highlighted in this review, fluency measures seem to be generally affected by several factors that go beyond disease stage and severity, including the educational and cognitive profile, as well as patients’ age (Obeso et al., 2012). This appears particularly crucial for action fluency tests, also due to task complexity and its relative unconstrained structure. In this respect, an approach focusing on individual differences will necessarily have to deal not only with more general compensatory mechanisms, but also with individual strategies that patients are likely to implement as action-related impairments become more prominent and impact more strongly their quality of life.

In line with a more detailed characterization of PD patients and their deficits, it is worth noting that the evidence on the possible neural correlates of performance in the action fluency test is so far still limited. However, the global assessment of action processing in PD will necessarily require assessing not only how behavioral impairments correlate with regional changes in brain activity, but also how compensatory mechanisms and medication are at play at different stages of the disease, as the preliminary research reviewed here has shown. Ideally, in this approach the comparisons between different PD subgroups (e.g., with and without dementia), should be extended to involve also other pathologies with different anatomical substrates and possibly behavioral manifestations of action processing impairments.

Finally, further attention should be devoted to how scores in the action fluency and similar tests/tasks is linked to the overall motor and communicative abilities of the patient, as this is likely to have a great impact on their quality of life. In fact, communication deficits in PD likely derive from a combination of motor and cognitive impairments (Smith and Caplan, 2018) but the actual impact of these deficits on daily communication, and thus the possible measures to mitigate and counteract them, is still poorly investigated and understood.

In conclusion, jointly addressing action semantic processing and motor dysfunctions in PD patients could pave the way to interventions where the motor deficits are addressed with to improve both motor and communicative skills since the early disease stages, with a likely significant impact on quality of life.

## Author Contributions

All authors listed have made a substantial, direct, and intellectual contribution to the work, and approved it for publication.

## Conflict of Interest

The authors declare that the research was conducted in the absence of any commercial or financial relationships that could be construed as a potential conflict of interest.

## Publisher’s Note

All claims expressed in this article are solely those of the authors and do not necessarily represent those of their affiliated organizations, or those of the publisher, the editors and the reviewers. Any product that may be evaluated in this article, or claim that may be made by its manufacturer, is not guaranteed or endorsed by the publisher.
